# Role for Cystathionine γ Lyase (CSE) in an Ethanol (E)-Induced Lesion in Fetal Brain GSH Homeostasis

**DOI:** 10.3390/ijms19051537

**Published:** 2018-05-22

**Authors:** Dhyanesh Patel, Marylatha Rathinam, Courtney Jarvis, Lenin Mahimainathan, George Henderson, Madhusudhanan Narasimhan

**Affiliations:** 1 Department of Pharmacology and Neuroscience, Texas Tech University Health Sciences Center, 3601 4th Street, Lubbock, TX 79430, USA; dhyanesh.patel@ttuhsc.edu (D.P.); mary.l.rathinam@ttuhsc.edu (M.R.); lenin.mahimainathan@ttuhsc.edu (L.M.); george.henderson@ttuhsc.edu (G.H.); 2 Department of Microbiology and Immunology, Texas Tech University Health Sciences Center, 3601 4th Street, Lubbock, TX 79430, USA; courtney.jarvis@ttuhsc.edu

**Keywords:** ethanol, fetal, brain, cystathionine, cysteine, glutathione, cysteine, CSE/CTH, transulfuration pathway

## Abstract

Earlier, we reported that gestational ethanol (E) can dysregulate neuron glutathione (GSH) homeostasis partially via impairing the EAAC1-mediated inward transport of Cysteine (Cys) and this can affect fetal brain development. In this study, we investigated if there is a role for the transulfuration pathway (TSP), a critical bio-synthetic point to supply Cys in E-induced dysregulation of GSH homeostasis. These studies utilized an in utero E binge model where the pregnant Sprague–Dawley (SD) rat dams received five doses of E at 3.5 g/kg by gastric intubation beginning embryonic day (ED) 17 until ED19 separated by 12 h. The postnatal day 7 (PN7) alcohol model employed an oral dosing of 4 g/kg body weight split into 2 feedings at 2 h interval and an iso-caloric and iso-volumic equivalent maltose-dextrin milk solution served as controls. The in vitro model consisted of cerebral cortical neuron cultures from embryonic day (ED) 16–17 fetus from SD rats and differentiated neurons from ED18 rat cerebral cortical neuroblasts. E concentrations were 4 mg/mL. E induced an accumulation of cystathionine in primary cortical neurons (PCNs), 2nd trimester equivalent in utero binge, and 3rd trimester equivalent PN7 model suggesting that breakdown of cystathionine, a required process for Cys supply is impaired. This was associated with a significant reduction in cystathionine γ-lyase (CSE) protein expression in PCN (*p* < 0.05) and in fetal cerebral cortex in utero (53%, *p* < 0.05) without a change in the expression of cystathionine β-synthase (CBS). Concomitantly, E decreased *Cse* mRNA expression in PCNs (by 32% within 6 h of exposure, *p* < 0.05) and in fetal brain (33%, *p* < 0.05). In parallel, knock down of CSE in differentiated rat cortical neuroblasts exaggerated the E-induced ROS, GSH loss with a pronounced caspase-3 activation and cell death. These studies illustrate the importance of TSP in CSE-related maintenance of GSH and the downstream events via Cys synthesis in neurons and fetal brain.

## 1. Introduction

The damaging effects of maternal ethanol (E) intake on the developing brain are extensively documented in animals and humans. Multiple brain regions (e.g., cerebellum, hippocampus, olfactory bulbs, and cerebral cortex) are vulnerable to E during development [1,2,3,4]. In vivo, clinically relevant blood E levels alter biochemical activity and molecular expression of several genes thereby compromising neurons in the developing brain and accelerating their rate of apoptotic death [4,5,6,7,8,9,10,11]. This enhanced apoptosis has been connected to the high sensitivity of fetal cells, especially a subpopulation of cells with the lowest GSH content to E-induced oxidative stress (OS) [1,2,3,4,5,6]. In general, OS in fetal environment resulting in alteration of the redox states of thiol disulfide “couples” such as reduced/oxidized glutathione (GSH/GSSG) and cysteine/cystine (Cys/CySS) has been one of the main mechanisms of teratogenesis [7,8,9,10]. GSH and thioredoxin (TRX) both regulate thiol modifications and maintain dithiol/disulfide redox environment essential to prevent cell transition to an orchestrated cell death mechanism. A key factor within the developmental setting is that these redox mechanisms must also be controlled temporally, which is likely affected by ethanol [1,3,11,12]. Normalizing GSH homeostasis prevented neuron death and attenuated the neurotoxicity [3,4,6,12]. Thus, the perturbation of GSH-based redox control and the associated OS may be critical driver of alcohol-induced molecular regulation of development and teratogenesis [4,13,14,15,16].

The transcription factor, nuclear factor E2-related factor 2 (Nrf2), is a master regulator of redox signaling that activates several antioxidant targets including but not limited to the genes governing GSH homeostasis. The latter include glutamate-cysteine ligase (GCL) (rate limiting enzyme in GSH synthesis), glutathione reductase (generation of reduced/active GSH), and multiple components of the gamma glutamyl cycle. Earlier, we have shown that E does upregulate Nrf2 in PCNs as a redox response [4]. But this increased Nrf2 response was not sufficient to retain normal GSH homeostasis thereby not preventing a specific population of fetal neurons from undergoing apoptosis [4]. However, the Nrf2 knockdown experiments revealed that the E-induced endogenous increase in Nrf2 is vital to cushion some of the alcohol-induced damage. Importantly, under the same condition when Nrf2 is ectopically overexpressed beyond its intrinsic level, it successfully prevented the redox impairment and neuronal vulnerability to alcohol [4]. Thus, we have recently addressed other cellular mechanism by which ethanol breach the Nrf2 defense and enable cellular injury in a neuronal population.

A potential explanation for this inadequate protection resides in kinetics of the synthesis of GSH. GSH, a tripeptide is synthesized de novo in a two-step enzymatically catalyzed reaction sequence [17,18] with the first reaction catalyzed by GCL. Importantly, the activity of GCL is dependent on its substrates, especially the Cys pool [19,20]. Thus, the homeostatic control of Cys availability is critical to maintenance of the physiological level of GSH synthesis [21]. One mode, by which a cell can generate Cys, should external sources be inadequate to maintain GSH homeostasis, is its synthesis by the trans-sulfuration pathway (TSP) [17,22]. Cys is synthesized from homocysteine by two enzyme systems, cystathionine β-synthase (CBS) and the rate-limiting cystathionine γ-lyase (CSE). Activities of these two enzymes are vital to maintenance of brain GSH [23,24,25]. The TSP has earlier been thought to only be active in the liver [18], but we now know that it is active in whole brain (including human), astrocytes and neurons ([22,24,25], and this report).

Since the disruption of glutathione homeostasis coupled with increased free radical load can enhance the sensitivity of alcohol to cellular apoptotic and growth processes during embryonic brain development, it is of central importance to define the mechanisms underlying GSH regulation so as to drive its reconstitution and rescue the impaired brain development process. Given the function of CSE in mitochondrial homeostasis, its absence and/or dysregulation can enhance oxidative stress and associated damage. This, coupled with CSE-based dysregulation has not been tested in the context of alcohol-induced neurodevelopmental toxicity. Thus, our goal in the current study has been to determine if there is a dysregulation of CSE and if so, is the impaired supply of Cysteine (Cys) due to CSE deregulation via trans-sulfuration pathway a/the control point for alcohol-induced GSH loss and cell death.

## 2. Results

### 2.1. Ethanol Increases Cystathionine Levels in Primary Cerebral Cortical Neurons (PCNs) and Fetal Brains

**Figure 1:**
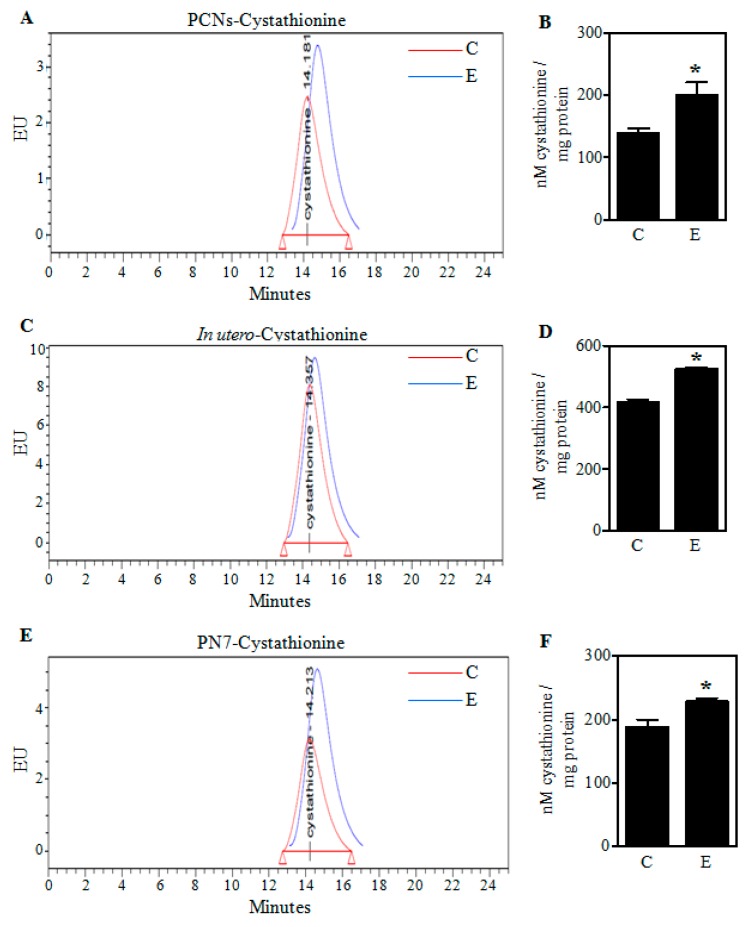
Effect of ethanol on cystathionine levels in PCNs and fetal brain cortices. A representative high-pressure liquid chromatography (HPLC) profile of cystathionine in Control (C) and ethanol (E)-treated PCNs (**A**); The concentration of cystathionine quantified using standards in PCNs (*n* = 6) (**B**); Pregnant rats (Sprague-Dawley) at embryonic day 17 (ED 17) were administered five doses of E (3.5 g/kg b.wt.) or isocaloric dextrose (Control) by gastric intubation at 12 h intervals. At ED 19 the brain cortex from embryos was dissected, the protein samples were HPLC analyzed for cystathionine. A representative cystathionine HPLC chromatogram of fetal brain cortices obtained from Control and E-exposed pregnant rats (**C**); Cystathionine concentration quantified with appropriate standards of the HPLC profiles of panel C (*n* = 3) (**D**); HPLC-based determination of cystathionine in brain cortices of postnatal day 7 (PN7) ethanol pups that received a oral dose of 4 g/kg body weight of 20% *v/v* ethanol (in milk solution) split into 2 feedings at 2 h interval with the controls receiving iso-caloric and iso-volumic equivalent maltose-dextrin milk solution substituted for ethanol (**E**); Fetal brain cortex cystathionine content quantification measured by HPLC in the PN7 model (*n* = 3) (**F**). Values represent the mean ± SEM. * *p* < 0.05 was considered significant vs. ethanol.

Earlier we have shown that E exposure decreases Cys and GSH content in both PCNs and binge alcohol model [26]. To gain a better understanding of the E-induced GSH loss and the role of transulfuration pathway (TSP), we first assessed the levels of cystathionine, an intermediate of TSP and an immediate precursor of Cys. Illustrated in Figure 1, both in vivo binge (2nd trimester equivalent) and PN7 model (3rd trimester equivalent) exposures and the in vitro PCNs treatment with E significantly increased cystathionine levels (*p* < 0.05). In particular, the increase was about 43%, 26%, and 22% in PCNs, in utero binge, and PN7 alcohol model respectively (Figure 1B,D,F).

### 2.2. Ethanol Decreases Cystathionine-γ Lyase (CSE) Protein in PCNs and Fetal Cortices of In Utero Binge Alcohol Exposed Rats

**Figure 2:**
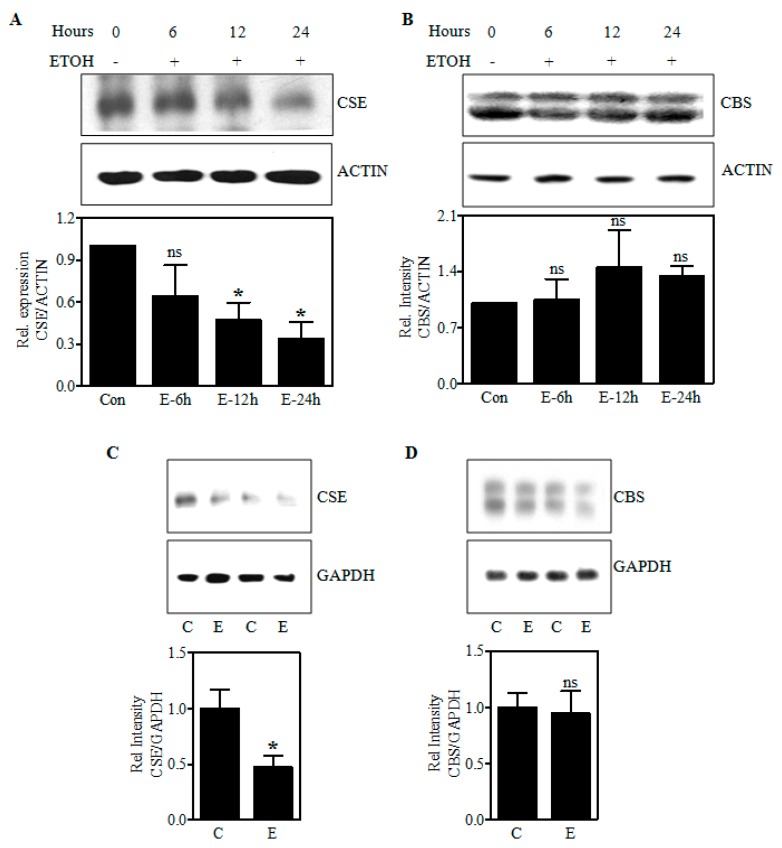
Effect of ethanol on CBS and CSE protein expression. A representative immunoblot image detecting CSE and ACTIN protein (**upper** panel) and its quantification (**bottom** panel) from Control and E-treated PCNs for indicated time points (hours) (*n* = 4) (**A**); Immunodetection of CBS and ACTIN protein in Control and E-treated PCNs measured by Western blotting (**upper** panel) and the corresponding quantification of CBS protein levels normalized to the reference protein, ACTIN (**bottom** panel) (*n* = 6) (**B**); Western blot image of CSE and glyceraldehyde-3-phosphate dehydrogenase (GAPDH) protein in fetal brain cortices obtained from Control or in utero E-exposed pregnant dams (**upper** panel) and the image densities of CSE relative to GAPDH (**bottom** panel) (*n* = 4) (**C**); Representative Western blots demonstrating the expression of CBS protein, with GAPDH as a loading control in fetal brain cortices of control and in utero alcohol-exposed pregnant rats (**upper** panel) and the quantification of immunoblots of CBS normalized to GAPDH (**bottom** panel) (*n* = 4) (**D**). Data are represented as mean ± SEM. * *p* < 0.05 was considered significant for ethanol.

In a setting of reduced Cys availability as shown by us earlier [26] with increased accumulation of cystathionine levels (in the current study), we next determined whether this might be caused by an altered expression of CSE, a rate-limiting enzyme that metabolizes cystathionine to Cys. Immunoblotting experiments for CSE revealed that CSE protein was reduced with the treatment of E in fetal PCNs by 36%, 53% (*p* < 0.05), and 64% (*p* < 0.05) at 6, 12, and 24 h, respectively. The protein levels of CBS, a multidomain enzyme that uses the cofactor pyridoxal phosphate and catalyze condensation of homocysteine and serine/cysteine to yield cystathionine [27] remained unchanged after E treatment in PCNs in relation to the ACTIN signal normalization (Figure 2B). Akin to the E-induced reduction in CSE protein in PCNs, the 2 days in utero E binge decreased the protein expression of CSE in fetal brain cerebral cortices by 53% (*p* < 0.05) (Figure 2C) with no change in CBS expression (Figure 2D). GAPDH signal was used to normalize the loading of the protein. These data suggest that ethanol can impair CSE protein expression likely leading to accumulation of cystathionine due to its poor utilization ultimately impairing Cys generation.

### 2.3. Ethanol-Induced Reduction of CSE Protein Is Associated with a Decrease in Its Transcript Levels

**Figure 3:**
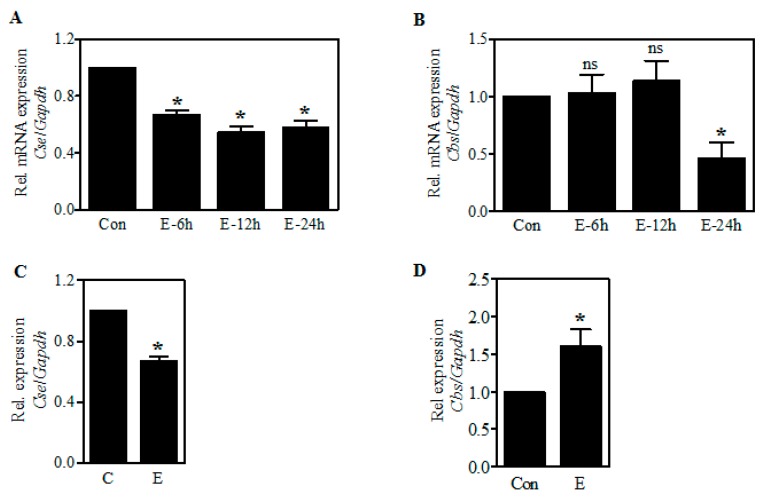
Effect of ethanol on the expression of *Cse* and *Cbs* mRNA. PCNs were treated with E (4 mg/mL) for the periods indicated and real-time qRT-PCR analysis for Cse transcript expression was performed. The fold change expression of *Cse* was determined by normalizing with the expression of a housekeeping gene, *Gapdh* (*n* = 4) (**A**); Real-time qRT-PCR based mRNA expression of *Cbs* gene relative to *Gapdh* from control and E-treated PCNs (*n* = 4) (**B**); Fetal brain cortices obtained from 2-day binge administration of iso-caloric dextrose and ethanol (as in Materials and Methods) was subjected to real-time qRT-PCR analysis for *Cse* mRNA expression *(n* = 5) (**C**). Real-time qRT-PCR analysis of *Cbs* gene relative to *Gapdh* from fetal brain cortices obtained from 2-day iso-caloric dextrose and ethanol-administered pregnant dams (as in Materials and Methods) (*n* = 5) (**D**). Values represent the mean ± SEM. * *p* < 0.05 was considered significant for ethanol alone.

To determine whether the effect of E on CSE occurs at the transcriptional level, we next performed real-time qPCR analysis for *Cse* mRNA expression using RNA extracted from control as well as E-treated PCNs and in fetal cerebral cortices exposed to E in utero. As shown in Figure 3A, the expression of *Cse* transcript was significantly reduced by 33% (*p* < 0.05) upon E treatment as early as 6 h in PCNs. This was further reduced to 45% (*p* < 0.05) and 51% (*p* < 0.05) at the end of 12 and 24 h exposure, respectively (Figure 3A). Similar to the CBS protein expression, the levels of *Cbs* mRNA at 6 and 12 h of E exposure remained unchanged with a significant decrease observed at the end of 24 h (*p* < 0.05) (Figure 3B). In parallel with the in vitro findings, a significant decrease by about 33% (*p* < 0.05) in the *Cse* mRNA expression occurred in the fetal brain neocortices from in utero binge alcohol exposed pregnancies (Figure 3C). However, in contrast to CBS protein expression, the *Cbs* mRNA was found to be significantly elevated (*p* < 0.05) in the fetal brain cortices obtained from the in utero binge exposed pregnant dams (Figure 3D). This implies that there is no tight concordance with *Cbs* mRNA and its protein expression in the in utero setting. It also supports the view that the transcription differences induced by E are somehow overridden by translational regulation such as delayed and/or inhibited protein synthesis, which gestational alcohol is known to cause [28]. Overall, these results suggest that ethanol-induced CSE dysregulation could occur either at the level of transcription or post-transcription processing.

### 2.4. Differentiation of Rat Cortical Neuroblasts to Neurons and Inhibition of CSE Expression by Small Interfering RNA (siRNA)

**Figure 4:**
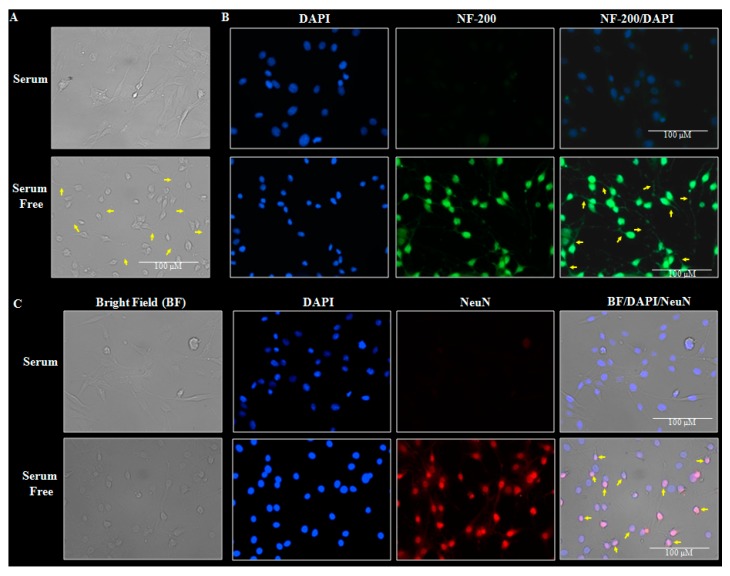

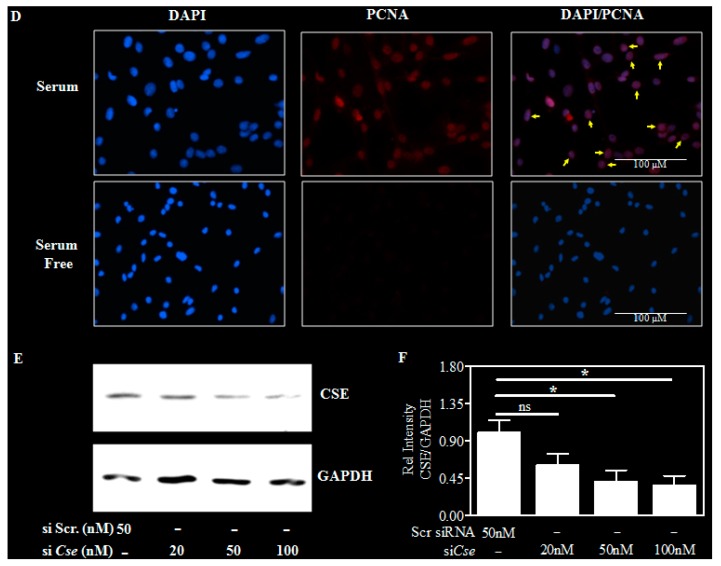
Differentiation of neuroblasts into neurons and siRNA-based downregulation of CSE in neuroblasts. A representative phase contrast microscopy 40× images of ED18 neuroblasts grown with and without serum and the yellow arrows indicate lengthy neurite outgrowth in serum free-exposed neuroblasts (*n* = 4) (**A**); The cells grown as in panel A were immunostained with antibody against neuronal marker, neurofilament-200 (NF-200) after 48 h and counter-stained with nuclear dye, DAPI. The cells positive for NF-200 (Green stain) indicate neuritogenesis (yellow arrow) and the DAPI (blue) indicate nuclei (*n* = 4) (**B**); Illustrative 40× immunofluorescent images of the cells maintained as in panel A stained with an antibody to the widely used neuronal nuclei marker, Neuronal Nuclei antigen (NeuN). Nuclear DNA was labeled with DAPI (*n* = 10). The yellow arrowheads in the merged panel (BF/DAPI/NeuN) indicate the neuronal nuclei that are double-positive for fluorescent staining of DAPI (blue) and NeuN (red) (**C**); Fluorescent images of immunofluorescent staining showing cortical neuroblasts under serum conditions show ready expression of the proliferation marker, proliferating cell nuclear antigen (PCNA) (red stain in the top panel), which was lost when differentiated (the bottom panel). The yellow arrowheads in the PCNA/DAPI merged panel indicate the proliferating/undifferentiated neuroblasts nuclei that is double-positive for fluorescent staining of DAPI and PCNA (*n* = 4) (**D**); A representative immunoblot image of CSE and GAPDH protein at the end of 48 h of transfection with the indicated concentrations of either non-targeting scramble siRNA or a SMARTpool mix of four si*Cse* in serum free media (**E**) and the densitometric quantification of CSE band relative to GAPDH (*n* = 3) (**F**). Values represent the mean ± SEM. * *p* < 0.05 was considered significant. In panels **A**–**D**, the scale bar indicates 100 μm.

Owing to the relatively low transfection efficiency and higher sensitivity of the primary neurons to culture conditions, we performed CSE loss-of-function experiments in a spontaneously immortalized cortical neuronal progenitors (neuroblasts) possessing inherent characteristics of proliferation and ultimately differentiating into post-mitotic cortical neurons. To this end, first we validated the efficiency of rat cortical neuroblasts isolated from ED18 fetus to undergo differentiation. Figure 4A illustrates the bright field image of the cortical neuroblasts grown under serum (proliferative) and serum free condition (differentiation). It is clear from the figure that the serum free-exposed neuroblasts reveal a clear lengthy neurite outgrowth, a characteristic of differentiation. Since, the neurofilament isoforms, a marker for maturing neurons that project neurites or processes increases as the development progresses [29,30], we next validated the differentiated neuroblasts with immunofluorescence for NF-200 expression. NF-200 expression was evident only in the serum-free exposed cells indicating the neuronal phenotype (Figure 4B). As, the neuronal nuclear protein (NeuN), is believed to express during early embryogenesis in postmitotic neuroblasts and continue to express throughout the differentiation and terminal differentiation process [29,31], we next determined the NF-200 immunostaining. NeuN, a protein that is predominantly associated with cell nuclei is present only in the serum-free exposed neuroblasts and not in the undifferentiated neuroblasts (yellow arrow, Figure 4C). Further, Proliferating cell nuclear antigen (PCNA), a marker to indicate proliferating neuroblasts [32] were expressed in cells grown in serum conditions and not the differentiated cells (yellow arrowheads; Figure 4D top panel). Having confirmed the efficiency of neuroblasts to differentiate into neurons, we next silenced CSE by transfecting the cells with either nontargeting scramble siRNA or a SMARTpool mix of four siRNAs against *Cse* along with serum free exposure. At the end of 48 h, the immunoblotting analyses for CSE clearly indicated a dose-dependent downregulation of CSE protein with a maximal ~70% downregulation at 100 nM concentration of siRNA that was chosen for the further experiments (Figure 4E,F). Together, these results indicate that the cortical neuroblasts can be differentiated into neurons and *Cse* can be efficiently silenced.

### 2.5. Cse Knockdown Reduces Neuron GSH and Potentiates Ethanol-Mediated GSH Depletion

**Figure 5:**
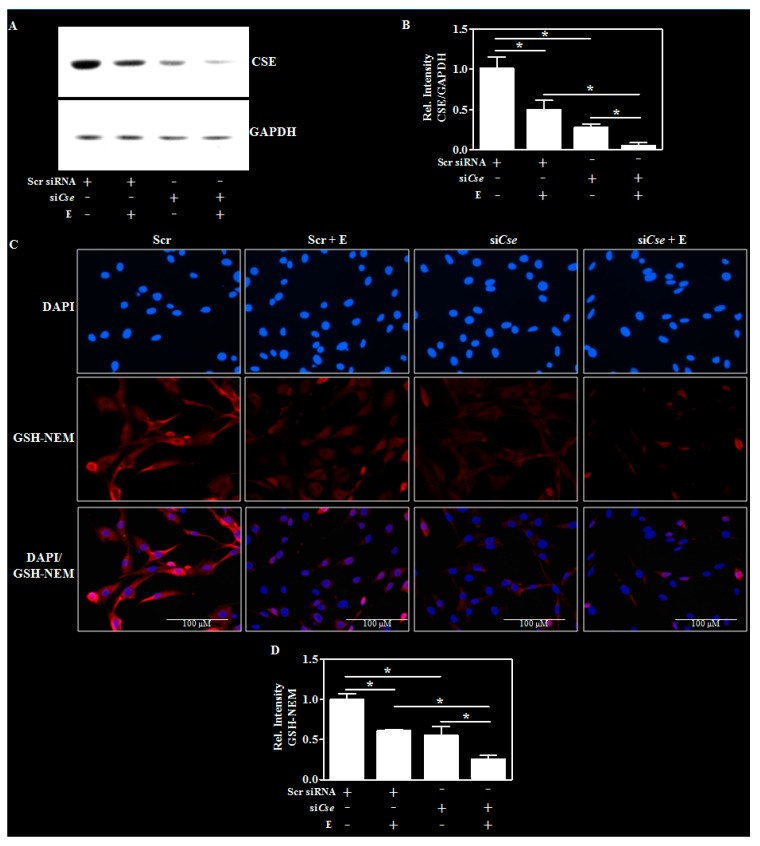
Effect of *Cse* knockdown on the E-induced glutathione changes in differentiated neurons. The transfection and the differentiation procedures were performed as in the Figure 4E except for the addition of E (4 mg/mL) after 24 h of transfection for an additional period of 24 h. A representative Western blot image of CSE and GAPDH (**A**) and the quantification of CSE protein relative to GAPDH (*n* = 3) (**B**); Cells were transfected and treated as in panel A and NEM was added to generate NEM-GSH adducts during permeabilization with methanol. Cell were then immunostained with antibody for GSH-NEM (red). The slides were counterstained with DAPI to identify nuclei (blue) and the images were captured at 40× magnification, with the scale bar indicating 100 μM. The specificity was validated by an absence of GSH-NEM signal in cells treated with NEM (data not shown) (**C**); Quantification of the neuronal GSH-NEM immunolabeled signals by Image J analysis (Bethesda, MD, USA) (**D**). Values represent the mean ± SEM. * *p* < 0.05 was considered significant.

Previous studies have shown that an inhibition of *Cse* resulted in lowered GSH levels in blood and brain [25] and to this end, we next tested whether silencing *Cse* affects and/or exaggerate E-induced GSH reduction. In addition to significantly decreasing the basal CSE protein content, the RNA interference-mediated silencing of *Cse* more robustly declined the E-induced CSE reduction (Figure 5A,B; lane 2 vs. lane 4). Next, we evaluated the changes in GSH content by adapting an immunohistochemical approach that uses antibody to glutathione-*N*-ethylmaleimide (GSH-NEM) adducts [33]. This method identifies GSH in NEM-treated samples eliminating the restricted specificity of antibodies against native GSH [34]. Interestingly, the enhanced knockdown of CSE protein elicited a similar exaggerated E-induced GSH decline by ~40% (*p* < 0.05) (Figure 5C,D—lane 2 vs. lane 4) indicating that CSE loss may be an important mechanism underlying the E-induced dysregulation of GSH homeostasis.

### 2.6. Effect of Cse Silencing on Ethanol-Mediated Reactive Oxygen Species (ROS) induction and Cell Death

**Figure 6:**
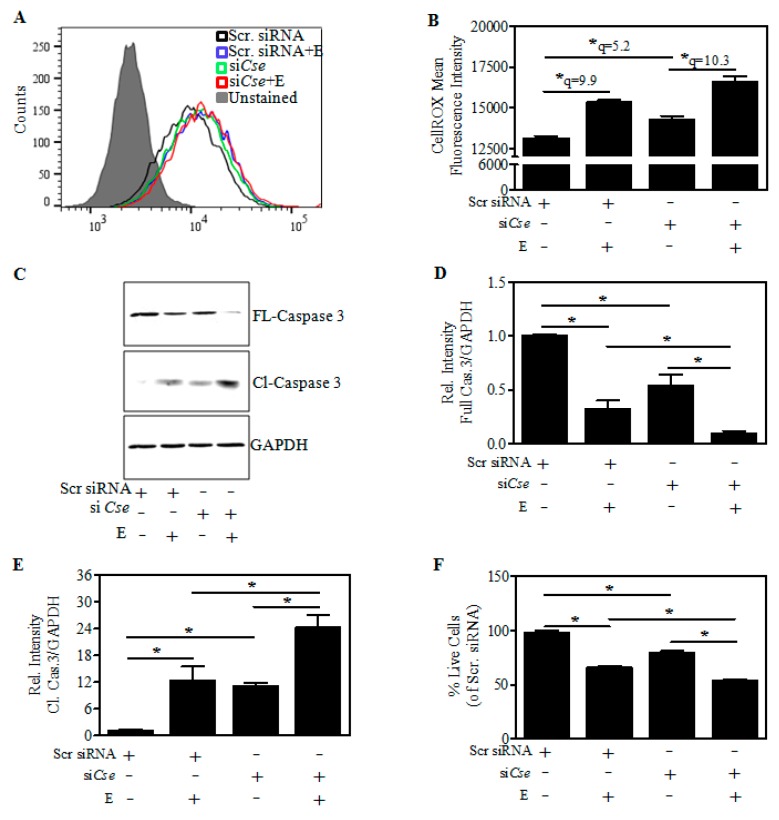
Effect of *Cse* knockdown on the E-induced cellular ROS changes and associated death of differentiated neurons. Transfection and the differentiation procedure were performed as in the Figure 5A. At the end of the experiment, the cells were stained with ROS-reactive dye, CellROX Green for 30 min and subjected to flow cytometry analysis. A representative Fluorescent-Activated Cell Sorting (FACS) histogram of scramble control (black line), scramble + E (blue line), si*Cse* (green line), si*Cse* + E (red line) stained with CellROX and an unstained control (filled grey) (**A**) and the quantification of CellROX-Mean fluorescent intensity (*n* = 3) (**B**); Cells were transfected and treated as in panel A followed by Western blotting. A representative immunoblot of the full length/inactive (FL) and cleaved/active (Cl) caspase-3 with GAPDH as a loading control (**C**); Densitometric quantification of the FL-caspase-3:GAPDH (*n* = 3) (**D**) and Cl-caspase-3:GAPDH was shown as the relative intensity of Scramble siRNA control (*n* = 3) (**E**); Cells were transfected and treated as in panel A and cell viability was determined by MTT assay. The result was shown as the percentage of scramble siRNA control (*n* = 6) (**F**). Data are represented as the mean ± SEM. * *p* < 0.05 was considered statistically significant.

As reduction in GSH can alter the redox balance, we next examined whether the exaggeration of E-induced GSH reduction upon *Cse* knockdown is accompanied by an exacerbation of the ROS load. As indicated in the representative histogram, an increase in the ROS levels was indicated as a shift of CellROX-Green fluorescence to the right after *Cse* silencing (Figure 6A; green vs. black line; Figure 6B; lane 1 vs. lane 3). Further in correspondence with E-induced GSH reduction, the E-induced increase in ROS was more robust in *Cse*-silenced cells (Figure 6B; lane 3 vs. lane 4). These data indicate that decreased CSE levels further increased the E-induced measures of cellular ROS. Since ROS can mediate apoptotic neuron death through caspase activation, we next determined the activation of caspase 3, a caspase that cleaves several substrates contributing to the apoptotic phenotype [35]. It is clear from the immunoblots that the full length (FL) caspase-3 (32 kDA), the inactive form is decreased upon E treatment and *Cse* knockdown in comparison to scramble transfected cells (Figure 6C) by ~60% and ~45% (Figure 6D). While the E-induced reduction in the pro-form of caspase 3 is further reduced significantly upon *Cse* knockdown (*p* < 0.05) (Figure 6D; lane 2 vs. lane 4). In contrast to the FL-caspase 3, the cleaved (Cl) caspase-3 (13 kDA), an active form, is significantly increased upon E and *Cse* knockdown (*p* < 0.05) (Figure 6C; lanes 2 and 3 vs. lane 1) which was pronounced in the E-exposed *Cse*-silenced neurons (Figure 6E; lane 2 vs. lane 4). In parallel with activation of caspase 3, the E-induced cell death of cortical neurons was also significantly exaggerated on *Cse* knockdown (Figure 6F; lane 2 vs. lane 4) collectively suggesting that *Cse* knockdown exacerbated the E-induced ROS, activation of caspase 3, and cell death.

## 3. Discussion

Redox impairment has been implicated in developmental neurotoxic and neurobehavioral abnormalities associated with gestational alcohol and there is compelling evidence that antioxidant manipulations could be utilized to mitigate damage to the developing brain caused by ethanol exposure. In particular, optimum levels of glutathione, a ubiquitous thiol antioxidant isrequired to maintain the redox status of the cells, including neurons, astrocytes, and neuronal progenitor cells [36,37]. An uncontrolled loss of GSH can exacerbate alcohol-induced neurotoxic responses. Although we have shown that dysregulation of GSH homeostasis along with apoptotic death of neurons occur in fetal brain and fetal neuron culture systems exposed to ethanol, our understanding of the mechanisms responsible for the decrease in GSH remain incomplete. Previously, we have shown that this E-impaired GSH homeostasis and death of neurons still occurred in the presence of E-mediated upregulation of Nrf2, a redox-responsive transcription factor that is involved in regulating the genes involved in GSH de novo biosynthetic pathway [4]. The current study for the first time demonstrates that a reduced expression of cystathionine gamma lyase (CSE), the rate-limiting enzyme for the synthesis of cysteine, and not the changes in cystathionine beta synthase (CBS) in primary cortical neurons (PCNs) and fetal brains exposed to prenatal alcohol may be a mechanism underlying the incomplete normalization of damaged fetal neuron GSH homeostasis by Nrf2. This was coupled with an accumulation of cystathionine, an indication of poor conversion to cysteine. Further, the inhibition of CSE in differentiated cortical neurons exacerbated the E-induced GSH loss which correlates with augmentation of E-induced ROS levels, activation of caspase 3, and ultimately the death of neurons.

A well-established cellular source of Cys for maintenance of GSH homeostasis is the transsulfuration pathway (TSP) which is a channel for the salvage of sulfur from the folate cycle. And within the pathway, CSE is a crucial generator of Cys [38,39] and studies are emerging that CSE and the TSP play a critical role in redox homeostasis in rodents and human brain [40,41]. While, the TSP has been extensively studied in liver, kidney, pancreas, heart and its role in brain has been debated. However, numerous reports have documented the presence of CBS and CSE in brain [22,23,24,25], yet not all have agreed as to specific localizations and relative tissue contents [22]. In particular, CSE activity was found to be almost similar in most regions of the brain, including cortex except for the hippocampus [40] and there is compelling evidence that CSE is present in neurons as well as glia [22,25,42]. Our present findings (Figure 2 and Figure 3) are consistent with this concept that CBS and CSE express in the intact fetal brain and fetal cortical neurons. Further, alcohol treatment decreased the expression of CSE protein in fetal brain cortices of binge alcohol exposed prenatal animals and PCNs exposed to alcohol without a change in the CBS expression, which supplies the CSE with cystathionine with serine and homocysteine condensation. A decreased CSE expression in the TSP could explain previously observed reductions of Cys and subsequent GSH synthesis with E exposure [26]. Consistent with this concept, we observed that alcohol caused a significant accumulation of cystathionine levels in the PCNs that was also replicated in the 2nd trimester equivalent in utero binge alcohol model and the 3rd trimester equivalent postnatal day (PN7) ethanol model as indicated by HPLC analysis. The resultant downstream impact of cystathionine accumulation on Cys and GSH levels were recently demonstrated by us [26]. Notably, previous reports in humans have shown that mutations of *Cse* gene causes an accumulation of cystathionine in liver, kidney, brain, and cerebrospinal fluid, leading to cystathionuria and decreased plasma cysteine concentration. This has been associated with mental retardation as well as mild to severe neurologic problems [43,44,45,46]. These experiments support the concept that the ethanol-mediated block of metabolic flux through the TSP occurs chiefly due to the lesion at CSE level and not in CBS. While, the mechanisms underlying E-induced CSE loss is incompletely understood at this time, our results on E-induced *Cse* transcript reduction could be attributed to one of or more of the mechanisms such as glucocorticoid signaling-dependent *Cse* gene regulation or miR-30-dependent post-transcriptional regulation of *Cse*, both of which is modulated by prenatal alcohol [47,48,49,50].

Interestingly, a previous report has shown a positive role of cystathionine in attenuating hepatic and kidney cell death by serving as a cysteine prodrug [51,52]. Moreover, during mammalian development, the brain tissue has high levels of cystathionine [53] and an apparent accumulation of cystathionine in human and primate brain is proposed to serve as cytoprotectant against neurotoxic insults and several neuropathological conditions, since a common underlying factor is the loss of cellular thiols in the form of GSH. Although the accumulation of cystathionine in the brain might be a reservoir of cysteine for GSH synthesis, if there is an imbalance between the relative levels and/or activity of CSE and subsequent cystathionine hydrolysis, as observed in the present study, cystathionine accumulation could reflect impaired Cys production. In the context of embryonic brain development such a setting can affect the timely processes that are reliant on the redox metabolism. Elevated cystathionine due to CSE loss and other conditions have been reported previously [54,55,56].

If an accumulation of cystathionine accompanied by a decrease in CSE is the cause for reduced cysteine and GSH levels in the alcohol treatment, knockdown of CSE would be expected to aggravate the E-induced GSH loss and result in generalized increase in ROS and enhanced cell death. Therefore, determining the role of CSE by the loss-of-function experiments is vital to our understanding of the overall TSP-associated downstream regulatory events on fetal neuron/brain GSH redox homeostasis. The technical difficulties of low transfection efficiencies with gene silencing using RNA interference strategies in primary cortical neurons are overcome by utilizing the rat cortical neuroblasts which can differentiate into cortical neurons. The immunohistochemical data demonstrated that the cortical neuroblasts can form neurites or processes and differentiate into new neurons (Figure 4) and we were able to consistently silence CSE in these cells by 70–75% (Figure 4). Using this, it was shown that E-induced CSE loss was further reduced in the CSE-silenced cells (Figure 5A,B). The CSE changes were precisely reflected in a significant reduction of basal GSH levels in CSE-silenced cells, which was potentiated when the CSE-depleted cells were exposed to ethanol (Figure 5C,D). This suggests the importance of CSE and intact TSP in maintaining the GSH homeostasis. This is consistent with previous studies using genetic knockdown of CSE expression or pharmacological inhibition of its activity in non-neuronal cellular and neurotoxic animal models which demonstrated that loss of CSE can produce a sustained loss of cellular total glutathione concentration, particularly of the reduced GSH [40,57,58,59].

A central outcome derived from this study is that the reduced CSE induced a profound impact on GSH loss that is correlated to an exacerbated oxidant load (Figure 6A,B). As previously shown, this redox imbalance resulted in activation of caspase 3 (Figure 6C,E) and predisposed the neurons to increased sensitivity to E-induced oxidative damage/cell death (Figure 6F). Thus, the current results indicate such mechanisms may be a resultant of E-induced CSE loss that fails to convert cystathionine to cysteine, an immediate precursor for the synthesis of glutathione, a master antioxidant. The absence of CSE causing oxidative shift coupled with growth retardation and an increased mortality has been shown in the non-neuronal models [23,60]. Further, there are several studies that have reported a deletion or inhibition of CSE and/or its activity as a critical factor in promoting neurotoxic, neurologic problems, and other pathogenic mechanisms [23,38,40,58]. While, it may be tempting to propose that the modulation of CSE expression and activity might be a therapeutic target in the settings of prenatal alcohol exposure, this is tempered by reports illustrating that in some conditions, CSE can be a mediator of inflammatory responses [61,62]. Given that CSE inhibition/absence and its outcome could vary with the type of disease and the targeted organ damaged, future work should address mechanisms of E-mediated CSE alterations and specific periods of deactivation/activation of CSE during specific stages of fetal brain development.

**Figure 7:**
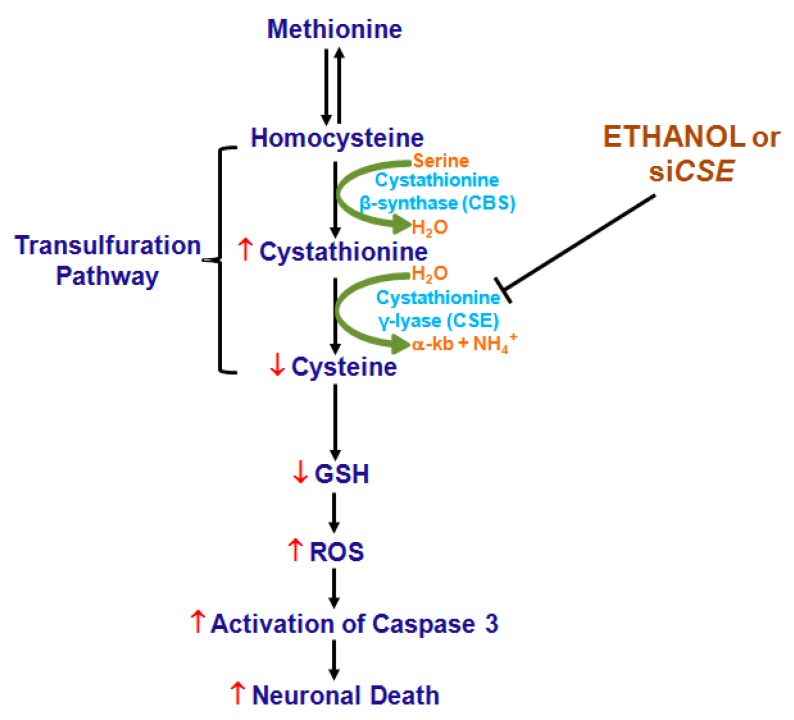
A proposed mechanism of alcohol-induced dysregulation of GSH. ↓ (red down arrow) and ↑ (red up arrow) indicates decreased and increased, respectively. H_2_O—Water, α-kb—Alpha-ketobutyrate, NH_4_^+^—Ammonium ion, 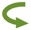 (green curved arrow) denotes the yielding reaction, ↓ (black down arrow) represents the downstream event(s) and ⊥ indicates inhibition/blockade.

In summary, this study illustrates that CSE loss and the resulting inefficiency in conversion of cystathionine to cysteine could be critical contributing factors responsible for the impaired GSH homeostasis-associated loss of cytoprotective signaling (Figure 7) in cellular and animal model of prenatal alcohol exposure.

## 4. Materials and Methods

### 4.1. Materials

Fetal bovine serum (FBS) and horse serum (HS) were purchased from Atlanta Biologicals (Lawrenceville, GA, USA). Minimum Essential Media (MEM), Ham’s F-12 medium, l-glutamine, penicillin-streptomycin and trypsin-EDTA were from Gibco (Grand Island, NY, USA). Protein assay reagent and polyvinylidene difluoride (PVDF) membrane were obtained from Biorad Laboratories (Hercules, CA, USA). HyBlot CL autoradiography film was obtained from Denville Scientific (Metuchen, NJ, USA). Smart Pool non-targeting siRNA and *Cse* siRNA pool were bought from Dharmacon Inc., (Lafayette, CO, USA). TriZol was bought from Invitrogen (Carlsbad, CA, USA). The antibodies used and their sources are as follows (Table 1).

FAM-dye labeled TaqMan gene expression assays for rat *Cse* (Rn_00567128), rat *Cbs* (Rn_00560948), and VIC-dye labeled TaqMan gene expression assay for rat *Gapdh* (Rn_01775763) were from Applied Biosystems (Foster City, CA, USA). CellROX-Green and SuperSignal West Pico chemiluminescence kit was bought from Thermofisher (Rockford, IL, USA). 3-(4,5-Dimethyl-2-thiazolyl)-2,5-diphenyl-2H-tetrazolium bromide (MTT), Triton-X 100, l-cystathionine and all other reagents were purchased from Sigma-Aldrich (St. Louis, MO, USA).

### 4.2. Primary Cortical Neuron (PCN) Cultures and Ethanol Treatment

PCNs were isolated from the brain of timed-pregnant, ED16-17 Sprague-Dawley rat as described [4,28]. Briefly, the fetal cortices were mechanically dissociated in HBSS and gently suspended in MEM containing 10% FBS and 10% HS. The cells were then seeded onto poly-d-lysine precoated-culture plates and were maintained at 37 °C in a humidified incubator containing 95% air and 5% CO_2_. 24 h post seeding, the cultures were treated with 10% HS supplemented MEM media containing 5-fluoro-2′-deoxy uridine (4 mg/mL) and uridine (10 mg/mL) to inhibit the growth of astrocytes and enrich neurons. Fresh media was replenished every 48 h and on the 5th day of culture, PCNs were subjected to E treatment. This primary neuronal culture model system from our laboratory consistently yields ~95% enriched neurons [4,28,63].

Primary cultures of cortical neurons were treated with 4 mg/mL E on day 5 in vitro (DIV). This concentration corresponds to 86 mM and it is a clinically relevant dose found in heavy drinkers [64,65]. Further, the E concentration used herein was shown to evoke an array of neurotoxic responses in various mouse and rat models [4,63,66]. In order to avoid the evaporation and maintain E concentrations in the culture media, all the E-treated plates were kept in an incubator that had been pre-saturated with alcohol for at least 24 h [28,30], while the control cells were maintained in the normal incubator.

### 4.3. Rat Brain Cortical Neuroblasts

The spontaneously immortalized rat brain neuroblasts obtained from cerebral cortices of 18-day fetal rats (E18 neuroblasts) used in this study were generously provided by Dr. Alberto Muñoz (Instituto de Investigaciones Biomédicas, CSIC, Madrid, Spain). These cells don’t express glia fibrillary acidic protein (GFAP), an astrocytic marker but are positive for NF-68 and primitive neuronal marker nestin [67]. Cells were cultured in Ham’s F-12 media containing 10% FBS, l-glutamine (2 mM), streptomycin (100 µg/mL), penicillin (100 units/mL) and plasmocin (5 µg/mL) and maintained in an incubator at 37 °C under an atmosphere of 95% air and 5% CO_2_. For differentiation and transfection experiments, the cells were grown in serum free media [30].

### 4.4. Small Interfering RNA (siRNA) Transfection

Cells were seeded in six-well plates at a density of 3 × 10^5^ cells/well. Following day, cells were transfected with either 20, 50 or 100 nM of siGenome smartpool mix of four *Cse* specific siRNAs or non-targeting siRNA pool were transfected into rat cortical neuroblasts in serum free condition using endoporter (Genetools, Philomath, OR, USA) [4,6,28,68]. Prior to transfection, cells were replaced with 800 µL of fresh media and transfected with 200 µL of transfection complex. CSE silencing was observed by Western analysis after 48 h. For the E treatment, 24 h after transfection of the 100 nM of respective siRNAs as mentioned above in the Section 4.2, 4 mg/mL of E was treated for additional 24 h and used in the respective downstream applications.

### 4.5. In Vivo Binge Model

A well-established 2-day in utero ethanol exposure rat model that mimics an alcohol binge exposure during 2nd trimester period in human pregnancy was used [2,4,28]. Briefly, Sprague Dawley rats were divided into two experimental groups and subjected to the following regimens: (1) Experimental group: This group received a total of five doses of ethanol (3.5 g/kg body wt, 25% *v*/*v*) every 12 h beginning on gestational day 17 with the final dose administered 2 h before sacrifice on gestational day 19 to maintain blood alcohol levels (2) Control group: Pair-fed and weight matched animals received five doses of iso-caloric dextrose instead of ethanol with all administration protocols remaining the same as with the experimental group. The solutions were delivered by intragastric gavage. All animals were maintained in accordance with Institutional Animal Care and Use Committee-approved procedures. Both isocaloric dextrose administered control and ethanol-fed dams had full access to water at all times. The pair-fed controls were given the same amount of standard chow consumed by their weight-matched ethanol dam during the previous 24 h period. The gestational age of the pair-fed control and ethanol rats were staggered by a day so as to offer the amount of chow eaten by the pair-fed control are at the same stage of gestation similar to the ethanol-treated female moms. At the end of treatment, dams were necropsied and blood was collected for alcohol analysis using Analox AM1 analyzer. The blood alcohol level was found to be between 2.5 ± 0.22 mg/mL (corresponding to ~55 ± 4.7 mM). Fetuses were surgically removed from the uterine horns and brain cortices were carefully isolated and stored at −80 °C until use.

### 4.6. In Vivo Postnatal (PN7) Model

A very well established postnatal 3rd trimester equivalent alcohol model was used as described [69,70]. Briefly, the day of birth was assigned as postnatal day 1 (PN1). Until the experimental day, PN7, the litters were kept with its respective dams. On PN7, body weights of the pups were determined, and the test group delivered a oral dose of 4 g ethanol/kg of 20% *v*/*v* ethanol (in milk solution) split into 2 feedings at 2 h interval. The control animals received gastric intubation of an iso-caloric and iso-volumic equivalent maltose-dextrin milk solution substituted for ethanol as described using small tubing lubricated in corn oil to overcome any resistance while swallowing the tube [69]. At the end of 4 h after the 1st dose, the brain cortices were collected from the neonates and 2 cortices were pooled into one and processed for HPLC based determination of cystathionine as in Section 4.7. Since the third trimester of pregnancy in rats to human equivalent is widely considered to occur postnatally between PN1-10 [71], and the timing of brain growth spurt was found to peak in PN7 in rats [72], we selected PN7 for our studies. 

### 4.7. HPLC Based Determination of Cystathionine

Assays of cystathionine utilized the HPLC method as described earlier with slight modifications [73,74]. Briefly, the metaphosphoric acid precipitated 100 µg protein sample was neutralized with saturated potassium carbonate and diluted with 0.2 M 1:4 borate buffer (pH 9.6). This diluted solution was derivatized with 0.2 M sodium borate buffer (pH 9.6) solution containing 15 mM *O*-phthaldialdehyde, 30 mM 2-mercaptoethanol, and 10% methanol for 1 min at 10 °C. An aliquot of the derivatized sample was then injected into the X-bridge C18 HPLC column and eluted at a flow rate of 0.8 mL/min with the mobile phase, 65% 0.1 M sodium acetate pH 4.75 and 35% methanol. The cystathionine chromatographic peak was detected at a retention time of 14.06 min at 340-nm excitation and 450-nm emission wave-lengths. The quantification of cystathionine was based on the peak height using the calibration coefficient obtained from the standard curve.

### 4.8. RNA Extraction and Real-Time qRT-PCR Analysis

Total RNA was isolated from PCNs or cerebral cortex using the TRIzol reagent according to the manufacturer’s recommendations (Invitrogen, Carlsbad, CA, USA). 1.5 µg of genomic DNA-eliminated total RNA was reverse-transcribed using the QuantiTect reverse transcription kit. A 20 µL real time RT-PCR reaction containing 1/10th of the cDNA, 10 µL of TaqMan Universal Master Mix (Applied Biosystems, Bedford, MA, USA), 20 pmol of the respective primer/probe mix was used to determine the *Cse*, *Cbs*, and *Gapdh* mRNA expression. The PCR cycling conditions included an initial denaturation step at 95 °C for 30 s followed by 40 PCR cycles at 95 °C for 5 s and 60 °C for 30 s. The expression of *Cse* and *Cbs* was determined relative to *Gapdh* as an internal control and the relative fold change in the mRNA expression was calculated using the 2^−ΔΔ*C*t^, where Δ*C*t = *C*t*_Cse (or) Cbs_* − *C*t*_Gapdh_* and ΔΔ*C*t = Δ*C*t_treated condition_ − Δ*C*t_untreated condition_.

### 4.9. Immunoblotting

PCNs or cerebral cortices were lysed in radio-immunoprecipitation assay (RIPA) buffer containing protease inhibitor cocktail (Sigma-Aldrich, St. Louis, MO, USA) on ice. The lysed homogenates were then sonicated (Sonics, vibra-cell ultrasonic processor) for 5 s and centrifuged at 15,000× *g* for 15 min at 4 °C. Total protein concentration was determined in the clarified lysates and equal amounts of cellular protein were loaded on 10% or 12% sodium dodecyl sulfate polyacrylamide gel and electrophoresed. The proteins were transferred onto a PVDF membrane and blocked in 5% nonfat dry milk powder (prepared in PBS with 1% Tween) for 1 h. The membranes were then incubated with primary antibodies against CSE, FL-caspase 3, Cl-caspase 3, GAPDH or ACTIN in 5% milk for either 3 h or overnight at 4 °C as previously described [28,68]. The primary antibody-incubated membranes were subsequently washed with PBST for 3 times, incubated with anti-rabbit IgG secondary antibody conjugated with horseradish peroxidase in PBST (1:5000 or 1:10,000) for 1 h at room temperature except for CSE blots that were incubated with the secondary antibody in 5% milk, washed with PBST for 5 min × 5 times each. The probed blots were subjected to ECL-chemiluminescence to detect the horseradish peroxidase. The protein signals were detected using SuperSignal West Pico chemiluminescence kit and captured onto an autoradiography film. The films were scanned using Adobe Photoshop CS2 (v9.0, Mountain View, CA, USA) at 300 dpi and the intensity of CSE, CBS, FL-caspase 3 or Cl-caspase 3 bands were quantified using NIH Image J and normalized to either GAPDH or ACTIN band intensity.

### 4.10. Immunofluorescence

Following the day of seeding, the rat cortical neuroblasts seeded in 8 well glass chamber slides (Lab-Teck II ThermoFisher Scientific, Rochester, NY, USA) were either allowed to grow in serum or serum free for additional 48 h. The cells were then fixed with 4% paraformaldehyde for 15 min and washed with PBS before permeabilization with 0.2% Triton-X 100 for 30 min. For GSH-NEM immunofluorescence, cells were transfected with 100 nM of siGenome smartpool mix of four Cse specific siRNAs or non-targeting siRNA pool with endoproter in serum free conditions for 24 h. Subsequently, cells were treated with 4 mg/mL of E in alcohol pre-saturated chamber for an additional 24 h. The chamber slides were then fixed with 4% paraformaldehyde for 15 min and washed three times with PBS. Subsequently, slides were incubated with 2 mM NEM in 100% methanol for 30 min to permeabilize cells and allow for the GSH-NEM adduct formation. This was followed by three PBS washes and blocking with 5% BSA for 30 min and appropriate primary antibody incubation (1:100) targeting NF-200, NeuN, PCNA or GSH-NEM overnight at 4 °C. The next day after PBS washes, the slides were incubated with secondary antibody (1:100) conjugated with Alexa flour 488 or 555 goat anti-rabbit/mouse IgG (H + L) for 3 h at RT in dark followed by three washes with PBS and mounted using DAPI containing mountant (Prolong Diamond Antifade Mountant with DAPI, Life Technologies, Eugene, OR, USA). Individual and overlay images were taken at 40× magnification with inverted digital fluorescence microscope (EVOS-fl, Fisher Scientific) under GFP or RFP channels for AF-488 or AF-555, respectively. Intensity of fluorescence signals captured from random fields was quantified using Image J software and expressed as relative fold change with respect to control.

### 4.11. Reactive Oxygen Species (ROS) Detection With CellROX-Green Reagent Using Flow Cytometry

The ROS detection was performed with CellROX-Green staining followed by flow cytometry analysis as per manufacturer’s recommendation. Briefly, at the end of the experiment CellROX-Green reagent at a final concentration of 2.5 μM was added to the cells and incubated at 37 °C for 30 min in a 5% CO_2_ humidified incubator. The cells were then harvested, washed once with phenol red free media, resuspended in PBS, and immediately subjected to flow cytometry on BD Accuri C6 flow cytometer (BD Biosciences, Franklin Lakes, NJ, USA). The fluorescence intensity was detected with a 530/30 nm band-pass emission filter and FL1 detector. The unstained cells gated using forward-scatter (FSC) and side-scatter (SSC) of light were used to exclude small debris and nullify the background fluorescence. The resulting FL1 data were plotted on a histogram. Data was collected by BD Accuri C6 flow cytometer (BD Biosciences) and analyzed with FlowJo v10.0.6 (FlowJo, LLC, Ashland, OR, USA).

### 4.12. MTT Assay

Cell viability was assessed using MTT assay. At the end of the incubations, the cells were gently washed with phenol red free RPMI-1640 media. MTT at a final concentration of 0.5 mg/mL was then directly added to the media and incubated at 37 °C for 3 h. The purple colored insoluble formazon crystals generated by viable cells were dissolved using dimethyl sulfoxide (DMSO). The absorbance was recorded at 560 nm with a reference at 750 nm in GloMax Multidetection System (Promega, Madison, WI, USA). The resultant 560 nm values corrected to 750 nM data were expressed as the percentage of viable cells relative to that of untreated controls.

### 4.13. Statistical Analysis

All data are presented as mean ± S.E.M. Experiments involving more than two groups were statistically analyzed using one-way analysis of variance (ANOVA) followed by Student–Newman–Keuls post-hoc comparisons and those that involve only two groups were analyzed using Student’s *t*-test. The analysis was carried out using GraphPad Prism software (Prism5, GraphPad, La Jolla, CA, USA). The *p* values less than 0.05 was considered as statistically significant.

## Figures and Tables

**Table 1:** List and source of antibodies.

S.No.	Antibody	Catalog #	Source
1	CSE	12217-1-AP	Proteintech, Rosemont, IL, USA
2	CBS	14782	Cell Signaling Technology, Beverly, MA, USA
3	PCNA	13110	
4	Cl-Caspase-3	9664	
5	Anti-rabbit IgG-HRP	7074	
6	GAPDH	sc25778	Santa Cruz Biotechnologies, Santa Cruz, CA, USA
7	NF-200	N4142	Sigma-Aldrich, St. Louis, MO, USA
8	Actin	A2066	
9	NeuN	MAB377	Chemicon, Temecula, CA, USA
10	GSH-NEM	MAB3194	Millipore Sigma, Burlington, MA, USA
11	FL-Caspase 3	ab4051	Abcam, Cambridge, MA, USA
12	Alexa flour 488 rabbit	A11008	Invitrogen, Carlsbad, CA, USA
13	Alexa flour 555 rabbit	A21428	
14	Alexa flour 555 mouse	A21422	

## Abbreviations

GSH	Reduced Glutathione
ROS	Reactive Oxygen Species
Cys	Cysteine
CSE	Cystathionine γ Lyase
CBS	Cystathionine β Synthase
E	Ethanol
ED	Embryonic Day
PCNs	Primary Cortical Neurons
PN7	Post-Natal Day 7
GSH-NEM	Glutathione-*N*-ethylmaleimide
TSP	Transulfuration Pathway
DAPI	4′,6-Diamidino-2-Phenylindole, Dihydrochloride
